# Do macrophages play a role in the adverse effects of endocrine disrupting chemicals (EDCs) on testicular functions?

**DOI:** 10.3389/ftox.2023.1242634

**Published:** 2023-08-31

**Authors:** Haoyi Cui, Martine Culty

**Affiliations:** Department of Pharmacology and Pharmaceutical Sciences, Alfred E. Mann School of Pharmacy and Pharmaceutical Sciences, University of Southern California, Los Angeles, CA, United States

**Keywords:** endocrine disrupting chemicals, testis, macrophages, germ cells, Leydig cells, genistein, DEHP, cell-cell interactions

## Abstract

During the past decades, several endocrine disrupting chemicals (EDCs) have been confirmed to affect male reproductive function and fertility in animal studies. EDCs are suspected to exert similar effects in humans, based on strong associations between levels of antiandrogenic EDCs in pregnant women and adverse reproductive effects in infants. Testicular macrophages (tMΦ) play a vital role in modulating immunological privilege and maintaining normal testicular homeostasis as well as fetal development. Although tMΦ were not historically studied in the context of endocrine disruption, they have emerged as potential targets to consider due to their critical role in regulating cells such as spermatogonial stem cells (SSCs) and Leydig cells. Few studies have examined the impact of EDCs on the ability of testicular cells to communicate and regulate each other’s functions. In this review, we recapitulate what is known about tMΦ functions and interactions with other cell types in the testis that support spermatogenesis and steroidogenesis. We also surveyed the literature for reports on the effects of the EDCs genistein and DEHP on tMΦ, SSCs, Sertoli and Leydig cells. Our goal is to explore the possibility that EDC disruption of tMΦ interactions with other cell types may play a role in their adverse effects on testicular developmental programming and functions. This approach will highlight gaps of knowledge, which, once resolved, should improve the risk assessment of EDC exposure and the development of safeguards to protect male reproductive functions.

## 1 Introduction

The most recent data from WHO reports a global infertility lifetime prevalence at 17.5% of the adult population worldwide (Infertility prevalence estimates, 1990–2021. Geneva: World Health Organization; 2023. Licence: CC BY-NC-SA 3.0 IGO). Among those, half of infertility cases are attributable to male factor (Agarwal et al., 2015). Concern has risen in recent decades about the increasing incidence of male reproductive disorders, including hypospadias and cryptorchidism in newborns, testicular cancer starting in adolescence, and a global decline in sperm counts and quality potentially linked to male infertility (Merzenich et al., 2010; Toppari et al., 2010; Agarwal et al., 2015; Barati et al., 2020; Levine et al., 2022). Testicular germ cell tumors (TGCTs) are the most common malignancies observed in young men from 15 to 44 years, including in USA where Hispanics are the most affected (Ghazarian et al., 2017). Studies have shown that early-life negative effects can lead to permanent changes in physiology and disease predisposition in adult life, which has been described as ‘developmental origins of adult disease hypothesis’ (de Boo and Harding, 2006; Arima and Fukuoka, 2020). This hypothesis also applies to diseases of the reproductive system, based on studies in which endocrine disrupting chemicals (EDCs) with antiandrogenic or estrogenic properties were found to affect sperm quality negatively and/or to be associated with male reproductive symptoms/disorders. Perinatal exposures EDCs are believed to play a major role in the etiology of these male reproductive disorders, classified under the umbrella term of testicular dysgenesis syndrome (TDS) (Skakkebaek, 2003; Skakkebaek et al., 2016). Overall, EDCs alter endocrine functions, body homeostasis, reproduction, and development by interfering with hormone function, synthesis, secretion, transport, metabolism, and elimination, exerting broad and long-term adverse effects (Diamanti-Kandarakis et al., 2009; Gore et al., 2015). EDCs that alter fetal androgens levels, androgen/estrogen balance or actions in the male reproductive tract, can impair the development and maintenance of male reproductive system during the masculinization programming window, resulting in TDS, as reported in the literature by us and others (Jones et al., 2014; Jones et al., 2015; Kilcoyne and Mitchell, 2019a).

A growing interest in the potential risk of exposure to EDCs and their association with adverse effects on infants, as well as children and adults has risen in recent years (Sun et al., 2013). Indeed, humans are subjected to multiple anthropogenic and natural EDCs from fetal life through adulthood (Diamanti-Kandarakis et al., 2009). Findings that fetal and neonatal exposures to EDCs in animal models result in a decline of male reproductive potential and genital birth defects have been substantiated in humans by epidemiological studies (Swan et al., 2005; Kalfa et al., 2015; Kilcoyne and Mitchell, 2019b). Although the current increased incidence of testicular cancer could relate to perinatal EDC exposure (Kristensen et al., 2008), it has not been experimentally demonstrated, due to most rodents models not developing testicular cancer, and to the difficulty of establishing strong associations between fetal/perinatal exposures to chemicals and a disease occurring later in life, in adolescent and young adults, who might have been exposed to many cancer-initiators, promoters and/or progressors in the years preceding the cancer diagnostic. Several studies have shown detectable levels of EDCs in breast milk, amniotic fluid, cord blood, urine and semen in humans (Adibi et al., 2009; Jones et al., 2014). Two common EDCs, the phytoestrogen genistein (GEN) and the plasticizer di-(2-ethylhexyl) phthalate (DEHP), and its bioactive metabolite mono-(2-ethylhexyl) phthalate (MEHP), are found in cord blood, amniotic fluid and breast milk (Jefferson and Williams, 2011; Lin et al., 2011; Wen et al., 2017). Fetal exposure to GEN was found to cause transient alterations in rat testes (Thuillier et al., 2009). Maternal exposure to GEN was reported to result in adverse effects on male reproductive system, including changes in spermatogenesis and steroidogenesis and reduced fertility in male offspring (Meena et al., 2017). Studies have also shown adverse effects of DEHP/MEHP on male reproductive system, including a decline in Leydig cell function, especially testosterone biosynthesis (Culty et al., 2008; Martinez-Arguelles et al., 2013).

The mammalian testis contains seminiferous tubules surrounded by the interstitium and performs two essential functions: spermatogenesis and steroidogenesis. The interstitial compartment comprises fibroblasts, blood vessels, leukocytes, mast cells, tMΦ, and steroidogenic Leydig cells. Spermatogenesis occurs in seminiferous tubules that contain germ cells (GCs) and somatic Sertoli cells (SCs), and are surrounded by peritubular myoid smooth muscle cells (PMCs) (Riccioli et al., 2006). EDCs can have differential effects on these various cell types, either by interfering with sex hormone homeostasis, by disrupting the factors they produce or by targeting cell-specific genes involved in critical interactions between cell types. The importance of these interactions is illustrated by the adverse impact of reduced testosterone production by Leydig cell on spermatogenesis. Similar to the central position exerted by Leydig cells on other testicular cell types, testicular macrophages (tMΦ) have been shown to interact with several cell types such as Leydig cells, but also spermatogonia, which rely on tMΦ for their survival and maintenance (DeFalco et al., 2015). However, there has been limited research on the effects of EDC exposures on such cell-cell interactions in testis, and more studies are needed to examine whether EDC deleterious effects could be due in part to the disruption of tMΦ interactions with other testicular cells. Understanding the impact of EDCs on tMΦ immunoregulation and cell interactions could shed light on some of the toxic mechanisms of action of EDC in testis. The present review addresses this possibility by (A) Providing a general overview of macrophages and their multiple functions. (B) Surveying what is known on tMΦ interactions with other testicular cells, with a particular focus on the role of tMΦ in the development and homeostasis of male reproductive system; (C) Review reported adverse effects of GEN and DEHP/MEHP, used as estrogenic and anti-androgenic prototypes, on tMΦ interactions with other testicular cell types.

## 2 The role of macrophages goes far beyond their innate immune functions

Macrophages are part of the body’s immune system, and secrete pro- and anti-inflammatory cytokines and chemokines, are ubiquitous and comprise two classes: resident macrophages and blood-borne infiltrating macrophages (circulating macrophages) (Liao et al., 2018). Studies have shown that most tissue-resident MΦ stem directly from yolk sac-derived erythromyeloid progenitors (EMPs) without monocyte intermediates and from fetal blood monocytes produced in the fetal liver (Liao et al., 2018). Another study has shown that circulating monocytes often arise from bone marrow progenitors and can derive from spleen under abnormal circumstances (during tumor development, or extramedullary hematopoiesis) (Qian et al., 2011). In the mouse, monocytes can be divided into two populations based on their Ly6C expression levels: Classical Ly6C^high^ monocytes and non-classical Ly6C^low^ monocytes (Honold and Nahrendorf, 2018). Classical Ly6C^high^ monocytes are derived from Ly6C^high^ bone marrow progenitors, mediated by chemokine receptor CCR2, while the non-classical Ly6C^low^ monocytes differentiate from classic Ly6C^high^ monocytes under the control of orphan nuclear receptor Nr4a17 (Serbina and Pamer, 2006; Hanna et al., 2011). Under healthy conditions, Ly6C^low^ monocytes exert a protective or anti-inflammatory effect during tissue injury or autoimmune joint inflammation (Misharin et al., 2014). “Classical” Ly6C^high^ monocytes can extravate and infiltrate tissues under homeostatic conditions to become resident MΦ, which have been shown to resolve tissue injury (Liao et al., 2018). Additionally, tissue-resident MΦ are differentiated immune cells that mature differently in particular tissues of the developing fetus, acquiring tissue-specific functional properties and changing their gene expression profiles accordingly (Hashimoto et al., 2013; Gomez Perdiguero et al., 2015; Song et al., 2017). Tissue-resident MΦ are long-lived, essential for tissue differentiation, physiology and homeostasis and can self-renew locally (Sieweke and Allen, 2013).

Tissue-resident MΦ, including tMΦ and monocyte-derived MΦ, are not only critical for tissue homeostasis, but they also play important roles in stress-induced responses and immune functions, including responses to infections and injury (Italiani and Boraschi, 2014; Italiani and Boraschi, 2017). Phagocytosis is an essential property of MΦ, supporting their role of “scavengers” to remove dead cells and other debris, important for healing processes (Seljelid and Eskeland, 1993). Given their rapid changes in accordance with different environmental stimulations, MΦ have been classified into classically activated (M1) and alternatively activated (M2) macrophages (Gordon, 2003; Martinez et al., 2009). M1 MΦ can be activated by interferon-γ (IFN-γ), lipopolysaccharide (LPS), granulocyte-macrophage colony-stimulating factor (GM-CSF) or other microbial stimuli, and can produce strong pro-inflammatory cytokines such as tumor necrosis factor (TNF α), Interleukin 1 beta (IL1b), and interleukin (IL 6) (Winnall et al., 2011). M1 MΦ produce nitric oxide (NO) to protect against bacteria and viruses and contribute to the clearance of microbial pathogens (Mosser and Edwards, 2008). In contrast, M2 MΦ are stimulated by IL-4, glucocorticoids, macrophage colony-stimulating factor (M-CSF), different inflammatory stimuli, and some immune complexes (Bode et al., 2012; Lee et al., 2014). M2 MΦ typically secrete anti-inflammatory cytokines, including interleukin (IL 10) or transforming growth factor (TGF β), and have distinct phenotypes (Gordon, 2003; Winnall et al., 2011; Sica and Mantovani, 2012). M2 MΦ can produce ornithine to promote proliferation and are associated with wound healing, tissue repair, parasite resistance, immuno-regulation. M2 MΦ are also able to attenuate the detrimental immune response (Sica and Mantovani, 2012). An important regulator of myeloid progenitors and monocyte and MΦ recruitment and differentiation that encapsulates well the broad role of MΦ beyond their immune function is Macrophage colony-stimulating factor (CSF1), a secreted cytokine exerting its action via binding on its receptor CSF1R, found to regulate non-immune cells expressing CSF1R, such as SSCs in testis (Stanley and Chitu, 2014; DeFalco et al., 2015).

## 3 Testicular macrophages and their interaction with other testicular cells

### 3.1 Characteristics of testicular macrophages

Testicular macrophages (tMΦ) are the most abundant immune cells in the mammalian testis (Mossadegh-Keller et al., 2017; Hosseini et al., 2022), where they play a critical role in controlling innate immune responses in infection and sterile inflammation via secreting pro-inflammatory and anti-inflammatory cytokines (Hedger, 2002). They can produce inflammatory responses to pathogens and bacteria by expressing major histocompatibility complex (MHC) class II antigens and Fc receptors in rodents (Rival et al., 2008). TMΦ can establish and maintain testicular immune privilege by protecting developing germ cells from immune assaults and preventing spermatogenesis from auto-immune assault and detrimental effects of autoimmunity (Mossadegh-Keller and Sieweke, 2018). In addition to their immune functions, tMΦ contribute to organogenesis, supporting fetal testis development and vascularization, the formation and normal functioning of SSCs, and tissue homeostasis (DeFalco et al., 2014; Potter and DeFalco, 2017; Mossadegh-Keller and Sieweke, 2018). Indeed, due to their immunosuppressive and testis-specific roles mentioned above, tMΦ are considered as the “guardians of fertility (Mossadegh-Keller and Sieweke, 2018). Two main subpopulations of tMΦ have been reported in rat testis: Resident testicular macrophages (Resident tMΦ) and Newly arrived testicular macrophages (Newly arrived tMΦ). This classification is characterized by the expression of a lysosomal antigen, CD68 (expressed in circulating monocytes and found in M1 and intermediary resident MΦ), and a scavenger receptor, marker, CD163 (expressed in tissue-resident M2 MΦ, and intermediary resident MΦ) (Table1) (Winnall et al., 2011; Winnall and Hedger, 2013). Resident M2 tMΦ are derived from precursor macrophages through local proliferation and were described as CD163+ tMΦ. TMΦ can also be distinguished by their localization either in the interstitium or at the periphery of the seminiferous tubules, defining different types of interactions with other types of testicular cells (Mossadegh-Keller et al., 2017; Potter and DeFalco, 2017). Besides newly-arrived tMΦ, defined as CD68^+^ CD163^–^ tMΦ that differentiated from infiltrated monocytes, other populations of tMΦ were identified as derived directly from bone-marrow progenitors, those derived from fetal BM progenitors forming interstitial tMΦ, whereas those derived from postnatal/prepubertal BM progenitors forming peritubular tMΦ (Hume, 2008; Winnall et al., 2011; Mossadegh-Keller et al., 2017). Studies have shown that tMΦ can produce high amounts of anti-inflammatory cytokine IL 10, as well as CXCL2, which is produced by both inflammatory and alternative tMΦ; and low amounts of pro-inflammatory factors TNF α and IL 1b when stimulated by LPS, IFN-γ in the case of pro-inflammatory tMΦ, or by IL 4 for alternative tMΦ (Winnall and Hedger, 2013). TMΦ maintain immune tolerance and the complex testicular microenvironment necessary for testis development and life-long testicular functions by communicating with other critical testicular cell types through cell-cell interactions via spatially associating with SSCs, SCs and Leydig cells (Skinner et al., 1991; Hales, 2002; DeFalco et al., 2014). Any disruption to these interactions could disrupt androgen synthesis and sperm production, potentially leading to hypogonadism-related disorders and male infertility (Skinner et al., 1991; Hales, 2002; DeFalco et al., 2014; Potter and DeFalco, 2017; Mossadegh-Keller and Sieweke, 2018).

**TABLE 1:** Origins, types, and main functions of testicular macrophages. Testicular macrophages can be distinguished by their origins, their polarization status, the cytokines they produce and their functions. The table summarizes the information collected from three references (Williams et al., 2018; Shi et al., 2022; Mass et al., 2023).

Origins	Age range	Macrophage polarization type	Main cytokines	Main function/targets
Yolk sac monocyte - fetal-derived macrophages	fetal/prenatal	M1	IL1b	Pro-inflammatory functions
			CD68	
			CXCL2	Regulate B cell differentiation, T cell antibody secretion, killing activity of natural killer cells (NK cells)
			CXCL10	
			IL1	
			IL6	
			IL12	Regulate the permeability of the blood-testis barrier (BTB)
			IL23	
			TNF-α	
			MCP-1	Regulate spermatogenic cell apoptosis
			NO	
Bone marrow monocytes - hematopoietic stem cells - common myeloid progenitor cells - monocyte-derived macrophages	postnatal/prepubertal	M2	TGFb	Anti-inflammatory and immune tolerance functions, Self-maintain
			IL10	
			CD163	

### 3.2 Cell-cell interaction between testicular macrophages and germ cells

The main interaction tMΦ have with germ cells is that with spermatogonia, which are in direct contact with the basement membrane of the seminiferous tubules. SSCs, the stem cells of the germline, are undifferentiated spermatogonia with pluripotent potential that form in early postnatal life (weeks 8–12 postnatal in human; ∼ PND6 in rodents) from their precursors, neonatal gonocytes (also called pre-/pro-spermatogonia) (Culty, 2013). In contrast to the first wave of spermatogonia which contribute only to the first round of spermatogenesis (Busada et al., 2016), SSCs sustain steady-state spermatogenesis after puberty, and as such they are essential to spermatogenesis and lifelong male fertility (Mei et al., 2015; Phillips et al., 2010). Similar to other stem cells, the SSCs have the ability to self-renewal and differentiate into other GCs, enabling the production and supply of sperm (Vlajković et al., 2012). Studies of SSCs transplantation in animals have shown an improvement of fertility, indicating that auto-transplantation of SSCs may be a promising therapeutic approach after fertility-ablative treatments in young males (Vlajković et al., 2012; Forbes et al., 2018; David and Orwig, 2020). Macrophage colony-stimulating factor (CSF1) is a secreted cytokine that has a significant role in the regulation of myeloid progenitor cells, and CSF1/CSF1R signaling is associated with the recruitment and differentiation of monocytes and macrophages (Stanley and Chitu, 2014; DeFalco et al., 2015). Interestingly, studies have shown that CSF1 promotes cell proliferation in primary cultures of isolated spermatogonia and SSCs, and that tMΦ express CSF1 that regulates the proliferation and differentiation of isolated type A spermatogonia and the spermatogonial-derived cell line C18–4 in culture, via local macrophage-spermatogonial interactions (DeFalco et al., 2015). The exact nature of this interaction does not seem resolved, but it could involve the diffusion of molecules secreted by tMΦ into adjacent SSCs in the intratubular space. The possibility that processes extending from peritubular tMΦ might directly contact SSCs would imply that the processes passed across the basement membrane. However, this was not observed in the study (DeFalco et al., 2015). *CSF1R* is expressed on both MΦ and SSCs. The role of CSF1/CSF1R signaling has also been shown to promote *in vitro* spermatogonial proliferation through the activation of mitogen-activated protein kinase (MAPK) (Kokkinaki et al., 2009). However, few studies have investigated the effects of EDCs on the interaction between tMΦ and spermatogonia. Besides the role of tMΦ in the maintenance of the spermatogonial niche, recent studies have identified the possible role of MΦ in regulating testicular germ cell tumors (TGCTs). A study examining the tumor microenvironment of metastatic vs. non-metastatic TGCTs reported increased levels of tumor-associated MΦ expressing Programmed death ligand 1 (PD-L1) in seminoma in comparison to non-metastatic TGCTs, proposing that PD-L1^+^ MΦ influenced the aggressive behavior of the tumors (Sadigh et al., 2020). Considering the established role of MΦ on SSC functions and their potential role on TGCT behavior, it would be interesting to explore the possibility that the disruption of tMΦ following early exposure to EDCs could contribute to the development or progression on TGCTs later in life.

### 3.3 Cell-cell interaction between testicular macrophages and Leydig cells

Steroidogenic Leydig cells, located between seminiferous tubules, produce and secrete testicular androgens, mainly testosterone, making them essential for the development of male reproductive tissues, sexual development, spermatogenesis and normal male reproductive function, (Zhou et al., 2019). Infertile men who are diagnosed with non-obstructive azoospermia (NOA) have hypertrophic Leydig cells with altered ultrastructure (Tash et al., 2002). Studies have shown altered production of testosterone and abnormal intratubular location of Leydig cells in men with cryptorchidism or Klinefelter’s syndrome (Regadera et al., 1991). Therefore, defective Leydig cell activity resulted in reduced testosterone production, increased follicle stimulating hormone (FSH) and luteinizing hormone (LH), impaired testis function, spermatogenesis failure, azoospermia and decreased fertility (Jezek et al., 1998; Joensen et al., 2008; Zirkin and Papadopoulos, 2018; Adamczewska et al., 2022).

The physical association between tMΦ and Leydig cells in the interstitium of the adult rat testis was first recognized in the late 1960s and extensively studied over the years (Christensen, 1969; Hutson, 1998). Morphological studies revealed this direct structural interaction via morphological contact sites between the membranes of macrophages and Leydig cells, known as “digitations” (Hutson et al., 1994). These digitations formed when interstitial tMΦ incorporated into clusters of Leydig cells upon puberty (Hutson, 1992), which is required for Leydig cell development and function during postnatal testicular maturation, as shown in macrophage-depleted transgenic csfm (op)/csfm (op) mice lacking CSF1 and in mice lacking macrophages after treatment with dichloromethylene diphosphonate (Gaytan et al., 1994a; Cohen et al., 1996; Cohen et al., 1997; Hutson, 2006). A study by Bergh found that the average volume density and total cell mass per testis of both macrophages and Leydig cells were reduced in cryptorchid testes (Bergh, 1985). This coordination of morphological changes of interstitial tMΦ and Leydig cells suggested that they were functionally related, further supporting a functional coupling between these 2 cell populations (Bergh, 1985; Bergh, 1987). In the rat testis, Leydig cells extended slender cytoplasmic processes to channels within an electron-dense network of adjacent macrophage membrane invaginations (Miller et al., 1983). It is believed that an intensive exchange of molecules and signals can occur in these channels between tMΦ and Leydig cells in both juvenile and adult testis through unique intercellular contacts or by secreting paracrine factors throughout development (Christensen, 1969; Miller et al., 1983; Hutson, 1992; Nes et al., 2000). Failure of this intimate communication between the 2 cell populations has been associated with azoospermia (Goluža et al., 2014). Moreover, the presence of tMΦ was necessary for rat Leydig cell differentiation in prepubertal rat and regeneration after ip injection of 75 mg/kg body weight ethylene dimethane sulfonate (EDS)-induced depletion in adult rats, whereas the ablation of tMΦ had no effect on Leydig cell number in intact adult testes (Gaytan et al., 1994a; Gaytan et al., 1994b). TMΦ and Leydig cells interact with each other through the secretion of cytokines and growth factors. Under normal physiological and non-inflammatory conditions, tMΦ secrete several essential cytokines, growth and differentiation factors to offer a proper microenvironment and extracellular milieu that regulates the development and differentiation of immature Leydig cells (Khan et al., 1992; Cohen et al., 1996; Cohen et al., 1997; Hales, 2002).

In inflammatory conditions, tMΦ are activated and provide several factors, including reactive oxygen species (ROS) and pro-inflammatory cytokines like interleukin-1 (IL-1) and tumor necrosis factor-alpha (TNF-α) to inhibit steroidogenesis in Leydig cells (Hales, 2002). However, excessive activation of tMΦ can cause chronic inflammation, even resulting in damaged Leydig cells, reduced testosterone production, and impaired testicular function. Moreover, tMΦ have been found to produce paracrine factors, such as 25-hydroxycholesterol (25-HC), acting as steroidogenic substrates for side chain cleavage, testosterone biosynthesis and steroidogenic functions in Leydig cells (Nes et al., 2000; Lukyanenko et al., 2001; Hutson, 2006; Singh et al., 2021). In turn, Leydig cells produce growth factors like insulin-like growth factor-1 (IGF-1) (Cailleau et al., 1990), which was found to be act as an anti-inflammatory cytokine (Nederlof et al., 2022) thereby may affect the inflammatory responses in tMΦ. An interesting local feedback interaction between mouse tMΦ and Leydig cells was recently reported, based on the *de novo* production of progesterone by tMΦ in mixed testicular interstitial co-cultures upon treatment with cAMP, known to be produced by Leydig cells, postulated to act on tMΦ via gap junction observed between the 2 cell types, whereas treating tMΦ with a M1-inducer mixture of LPS and interferon-γ inhibited progesterone synthesis by MΦ (Yamauchi et al., 2022).

Overall, the macrophage-Leydig cell interaction is a complicated process involving the secretion of several cytokines and growth factors. It is crucial for maintaining normal testicular steroidogenesis, Leydig cell function and male lifelong fertility, and disruptions in this unique interaction can result in testicular dysfunction and reduced fertility. Further research identifying the mechanism underlying the developmental and functional links between macrophages and Leydig cells could provide new insights into potential therapeutic options for male infertility.

### 3.4 Is there evidence of cell-cell interactions between testicular macrophages and Sertoli cells?

In contrast to spermatogonia and Leydig cells, which have well established interactions with tMΦ, to the best of our knowledge, there is no study demonstrating the existence of cell-cell interaction between tMΦ and Sertoli cells (SCs). SCs are the only somatic cells in the seminiferous epithelium, where they provide physical and nutritional support to the survival and differentiation of germ cells, starting in fetal testis and throughout mammalian spermatogenesis, via regulated cell junctions, cell-cell interactions, and biochemical components by secreting lactate, cytokines, and hormones. SCs also form an immune-protective environment for autoantigenic germ cells via physically inhibiting the invasion of harmful substances by the blood-testis barrier (BTB) and secreting immunomodulatory factors. Studies have also shown that SCs play a crucial role in the phagocytosis of the elongated spermatids’ cytoplasm by engulfing dead or dying spermatogenic cells (Shiratsuchi et al., 2013). These cell-cell interactions are important for the development, homeostasis, and regulation of male reproductive system (O'Donnell et al., 2000). Sertoli cells support, nourish, and protect spermatogenic cells by various signal pathways. The TGF-β/Smad, AMPK and MAPK signaling pathways are involved in immature Sertoli cell proliferation and in the dynamics of tight junctions and adherent junctions in mature Sertoli cells to support spermatogenesis. The MAPK and AMPK signaling pathways also regulate the lactate production of Sertoli cells to maintain normal carbohydrate metabolism of germ cells, while the MAPK signaling pathway also exerts a dominant role in regulating SSCs self-renewal. A recent study found that the activities of these signaling pathways were abnormal in the Sertoli cells of testicular cancer or infertile patients (Ni et al., 2019). In addition, mature Sertoli cells produce androgen-binding protein (Abp), transferrin, proteases, and protease inhibitors, all of which are essential for maintaining spermatogenesis, sperm maturation, the tight SCs junctions, and the seminiferous remodeling processes. Moreover, SCs can secrete growth factors, such as TGF‐β, PDGF, VEGF, stem cell factor, and express glycoproteins that are necessary for the structure of the basement membranes between the SCs and peritubular cells (Luca et al., 2018).

Although these studies do not demonstrate an interaction between tMΦ and Sertoli cells, there are several studies suggesting that Sertoli cells may indirectly participate to macrophages role in testis. Mouse and human Sertoli cells express CXCL12 (Chen et al., 2015; Westernstroer et al., 2015; Guo et al., 2018), facilitating the maintenance of CXCR4^+^ spermatogonia by producing CXCL12, the ligand of CXCR4, a receptor expressed in both human macrophages and spermatogonia (Guo et al., 2018). Taken together with the immunomodulator role of Sertoli cells, it is reasonable to think that both Sertoli cells and tMΦ may modulate or support their respective effects on testicular inflammation and spermatogenesis. A more direct interaction between the 2 cell types cannot be excluded. Therefore, clarifying the effects of EDCs on potential interactions between macrophages and Sertoli cells could contribute to a better understanding of their toxicological mechanisms of signaling pathways in male reproduction and provide novel insights into identifying potential therapeutic targets of reproductive disorders. More recently, hematopoietic lineage-tracing studies in mice depleted in Sertoli cells, using Sertoli-specific Amh-cre mice, revealed that Sertoli cells are critical in recruiting monocytes originating from fetal hematopoietic stem cells to become tMΦ in the developing gonads (Gu et al., 2023).

## 4 Comparison of GEN and DEHP/MEHP effects on macrophages and other testicular cells

### 4.1 GEN and DEHP/MEHP as EDC prototypes

EDCs include widely used man-made compounds used in the production of plastics, pesticides, lubricants, preservatives, fungicides, medical devices, and additives in consumer products such as cosmetics and personal care products, as well as natural substances, such as phytoestrogens that are found in food (Gore et al., 2015). Human exposure to EDCs has been well documented through multiple studies measuring the parent compounds and/or metabolites in human body, such as in semen, breast milk, amniotic fluid, and cord blood (Adibi et al., 2009). Moreover, many EDCs have high fat-solubility and low water-solubility facilitating their accumulation in body tissues (Diamanti-Kandarakis et al., 2009). Exposure to EDCs, particularly those with antiandrogenic or estrogenic properties, during the masculinization programming window, can perturb fetal programming and gonadal development, leading to testicular dysgenesis syndrome (TDS), as demonstrated in animal models, including our own studies (DiVall, 2013; Jones et al., 2014; Jones et al., 2015).

Genistein, the main phytoestrogen in soy, has extensive biochemical properties, in addition of its estrogenicity via binding to estrogen receptors (ERs), mainly ESR1 and 2 (Erα and Erβ), exerting also direct or indirect antioxidant action and activating peroxisome proliferator-activated receptor (PPAR), as well as inhibiting tyrosine kinases (Jones et al., 2015). Estrogens play an essential role in the regulation of testis development and the male reproductive tract, which express estrogen receptors and are responsive to estrogens from fetal life to adulthood (Lehraiki et al., 2011). Fetal exposure to genistein occurs mainly through maternal uptake of soy-derived products and then undergoes placental transfer. Soy-based formula-fed infants have high circulating GEN levels of up to 9 mg/kg/day, with urine concentration levels around 500 times higher than that of infants fed cow milk-based formula (Setchell et al., 1997; Cao et al., 2009). Studies have also measured GEN blood levels up to 10.2 µM in 4-year-old infants (Jefferson and Williams, 2011). By mimicking the structure of estradiol and binding to ERs at inappropriate times during development, GEN can produce adverse effects on male fertility (Cederroth et al., 2010). Studies have shown that fetal and neonatal exposures of rats to GEN induced transient changes in intracellular signaling (such as MAPK, PDGFR) in neonatal gonocytes and alterations in spermatogenesis (Thuillier et al., 2003; Wang et al., 2004; Thuillier et al., 2009).

Humans are chronically exposed to DEHP, the most used phthalate plasticizer, which leaches from plastics and other consumer products to which it is added but not covalently bound (Gore et al., 2015; Walker et al., 2021). Fetal exposure to DEHP is predominantly through maternal ingestion, dermal contact, or inhalation. Neonatal exposure to DEHP takes place mainly through ingestion by infants, such as drinking contaminated breast milk (Zhu et al., 2006). Studies have found measurable amounts of DEHP or its metabolites in human blood, amniotic fluid, cord blood, and urine (Calafat et al., 2004). In rodents, DEHP or its metabolites, including its main bioactive metabolite Mono-(2-ethylhexyl) phthalate (MEHP) have also been found in amniotic fluid and urine following ingestion (Calafat et al., 2006). DEHP and MEHP can produce androgen receptor-independent antiandrogen effects through biding on *PPAR*s, but have also been shown to alter DNA methylation status and affect estradiol-regulated proteins, all of which could impair testosterone biosynthesis in fetal and adult mice (Culty et al., 2008).

Several *in vivo* and *in vitro* studies have examined the effects of DEHP/MEHP and other phthalates on MΦs, using concentrations ranging from 10^−8^ to 10^−3^ M for *in vitro studies*, and from 1 to 1,000 mg/kg/day for *in vivo* and *ex-vivo* studies. These studies, including some using the human THP-1 cell line, reported variable effects of MEHP, from decreased to increased cytokine production and phagocytosis depending on the concentrations and macrophage cell lines used (Li et al., 1998; Bennasroune et al., 2012; Hansen et al., 2015). Few studies also tested the effects of genistein on MΦ cell lines, using concentrations from 50 nM to 100 µM and reporting mostly anti-inflammatory effects (Mueller et al., 2010). However, these studies did not address the possibility that exposure of MΦ to these chemicals could alter cellular networks and the biological responses of tissues such as the testis.

### 4.2 Can GEN and DEHP/MEHP alter macrophages and germ cells interactions?

Although there is no study reporting direct or indirect effects of GEN and DEHP/MEHP on MΦ and germ cells interactions, or on Csf1 signaling pathway, critical for MΦ-SSC interactions, the possibility can be inferred from studies describing the disruption of both cell types upon exposure to these EDCs. Caspase-3-positive immunostaining studies of human fetal testes demonstrated that MEHP treatment decreased germ cell number by inducing germ cell apoptosis *in vitro* (Lambrot et al., 2009). Previous work from our laboratory have shown that *in-utero* exposure of rats to 10 mg/kg/day of GEN and DEHP mixtures (GEN-DEHP) altered short- and long-term gene expression and protein levels of germ and somatic cell markers, and increased incidence of infertility in rats exposed to GEN-DEHP mix at this human-relevant dose, differently from exposure to individual EDCs (Jones et al., 2014; Jones et al., 2015). Additionally, *in-utero* exposures to 0.1 and 10 mg/kg/day of GEN-DEHP induced alterations in testicular macrophages, both in neonatal and adult rats, with transcriptome analyses pointing at genes and pathways uniquely altered by GEN-DEHP mixtures, while others were common between mixtures and single EDCs at the same low doses (Walker et al., 2020). Peroxisome proliferator-activated receptors gamma (PPAR g) is expressed in monocytes and MΦ, regulating fatty acid synthesis, glucose metabolism, and the immune inflammatory (anti-inflammatory) response (Chinetti et al., 2003). Moreover, PPAR g agonists have been shown to inhibit pro-inflammatory cytokines (TNF α, IL 1β, and IL 6) synthesis in a dose-dependent manner (Heming et al., 2018). Moreover, GEN was reported to impede CSF1 action in osteoclast homeostasis, providing a model in support of the possible disruption of MΦ-SSC interactions by GEN (Amano et al., 1998). Thus, we predicted that fetal tMΦs could be direct targets of GEN and/or DEHP in our *in-utero* exposure model, permanently disrupting their interaction with testicular cells such as spermatogonia after birth. However, our studies did not resolve the mechanisms by which the two EDCs affected mechanisms underlying these interactions and spermatogonia postnatally, and whether such effects could be triggered by postnatal exposures.

In view of these studies, one can reasonably predict that the perturbation of MΦ-SSC interactions may contribute to the adverse effects of EDCs on spermatogenesis and male fertility. For example, considering the key role played by tMΦs-produced CSF1, if *in vivo* exposure to GEN-DEHP mixtures (or other EDCs with equivalent mechanism of action and molecular targets) inhibited the production of CSF1, disrupting its signaling on SSC-expressed CSF1R and altering the tyrosine kinases downstream of CSF1R, which could result in inadequate SSC pool, failure of spermatogenesis and infertility, as observed in ours and others animal studies of EDC exposures. It is therefore important to investigate the biological effect and molecular mechanisms of GEN, DEHP/MEHP and their mixtures on immune and inflammatory processes and spermatogenesis.

### 4.3 Can GEN and DEHP/MEHP alter macrophages and Leydig cells interactions?

A number of studies have shown that Leydig cells are direct targets of EDC mixtures (Svechnikov et al., 2010; Walker et al., 2021), and that EDCs can disrupt steroidogenesis in prepubertal Leydig cells (Akingbemi et al., 2004; Li et al., 2013; Yawer et al., 2020). In prepubertal Leydig TM3 cells, EDCs have been shown to affect the expression of gap junctional protein connexin 43 (Cx43) and intercellular signaling (Yawer et al., 2020). Consistent with this finding, a recent meta-analysis of the literature on the effects of isoflavone on Leydig cells found that GEN, administered during the perinatal period to mice at doses ranging from 50 to 1,000 mg/L or 10–600 mg/kg, depending of the studies, exerted overall adverse effects on Leydig cells, downregulating gene expression, alter protein-protein interactions, interfering with androgen biosynthesis and luteinizing hormone-dependent signaling and reducing the protein expression of adiponectin and AdipoR2, suggesting an impaired endocrine function (Hancock et al., 2009; Lozi et al., 2021). In Leydig cells, both GEN and MEHP individual exposures are reported to decrease the gene expression of steroidogenic acute regulatory protein StAR (Enangue Njembele and Tremblay, 2021; Lozi et al., 2021). DEHP and MEHP exposures both decreased cell viability and steroidogenic potential in mouse MA-10 cells (Piché et al., 2012). Microarray analysis of cultured rat fetal testis revealed that MEHP altered the gene expression of Cyp17a1, which is involved in Leydig cell steroidogenesis, while reducing testosterone production in a dose-dependent manner (Chauvigné et al., 2011). DEHP was found to have a deleterious impact on cell proliferation and testosterone production during development (Ge et al., 2007). In our own studies, *in utero* exposure to various doses of DEHP was found to reduce both fetal and adult Leydig cell testosterone production in rats, with concomitant decrease in steroidogenic enzymes in fetal testis, but not in the adult, leading to the subsequent discovery of an additional effect of fetal DEHP exposure on testis via altered aldosterone production in adrenal glands in the adult (Culty et al., 2008; Martinez-Arguelles et al., 2011). Adding 10 µM MEHP to organ cultures of fetal rat testes treated with the LH analog hCG reduced testosterone production (Boisvert et al., 2016), similarly to the *in vivo* effects of DEHPs. However, when PND3 rat testis were used instead of fetal testes, the production of basal, but not hormone-induced testosterone, was increased by 10 µM MEHP (Jones et al., 2015). In the same study, co-treatment of MEHP and GEN brought down basal testosterone to control level, indicating that GEN countered MEHP stimulatory effects on Leydig cells. Interestingly, treating mouse tumoral MA-10 Leydig cells with 10 μM GEN-MEHP mixtures increased steroid production in basal condition, but decrease it the presence of hCG, further indicating that Leydig cells may respond differently to EDCs according to their hormonal status (Jones et al., 2016). Concurrently, 10 μM GEN-MEHP mixture altered lipid profiles and decreased Insl3 mRNA, a functional marker of Leydig cells, suggesting endocrine disruption and Leydig cell dysfunction (Jones et al., 2016). Recent studies suggested that the pro-inflammatory effects of tMΦ may affect testosterone synthesis and metabolic processes in Leydig cells (Harris et al., 2016; Ye et al., 2021; Gabriela et al., 2022). These results imply that Leydig cell functions can be impacted by testicular macrophages responses to EDC mixtures under inflammatory conditions. Furthermore, a study performed on 514 mothers and their infants (Hokkaido Study Sapporo Cohort Study) reported that maternal blood levels of DEHP and MEHP were associated with hormonal changes in their infants blood, including altered testosterone/estradiol ratio, progesterone levels, and Insulin like 3 (INSL3) levels, indicative of Leydig cell functional disruption in the infants (Araki et al., 2014).

All together, these studies support the possibility that the disruption of Leydig cell-tMΦ play a role in the adverse effects of EDCs on testicular function. However, this has been poorly investigated. Understanding the impact of EDCs on the signaling pathways involved in macrophage-Leydig interactions and the mechanisms underlying these interactions should help us gain insight into the risk of such exposures on male reproductive functions.

### 4.4 Are there common threads between GEN and DEHP/MEHP effects on macrophages and Sertoli cells?

To our knowledge, there is no study examining the effects of DEHP/MEHP on tMΦ and Sertoli cell interactions, but there are studies reporting effects of these EDCs on Sertoli cells. Early studies reported that DEHP induced morphologic changes in prepubertal Sertoli cells, and to inhibit cAMP accumulation and intracellular ATP levels in immature Sertoli cells by changing FSH receptor signaling in rats (Creasy et al., 1983; Heindel and Powell, 1992; Richburg and Boekelheide, 1996; Lee et al., 1997). DEHP treatment increased the mRNA expression of PPARα and PPARγ but decreased the protein levels of phosphorylated mitogen-activated protein kinase (activated MAPK) in Sertoli cells (Bhattacharya et al., 2005). In another study, exposure of pregnant mice to 2 mg/kg/day DEHP was found to disrupt the differentiation of fetal Sertoli cells and subsequent postnatal immature Sertoli cells by altering the regulation of sex determination genes (Wang et al., 2016). In addition, treating mice from conception to lactation with a diet containing 40 mg/kg GEN increased seminiferous tubule diameter and testosterone production, whereas treatment with 800 mg/kg GEN, a dose exceeding human exposure levels, reduced seminiferous tubule diameter and decreased the gene expression of the Sertoli cell marker SOX9, indicating opposite effects of GEN on Sertoli cells depending of the doses, and Sertoli cell toxic effects for the high GEN concentration (Shi et al., 2020). In our own *in utero* exposure studies using the human-relevant dose of 10 mg/kg/day GEN and DEHP, we found that Abp gene expression was reduced in juvenile rat Sertoli cell by DEHP and GEN-DEHP, while Amh and Wt1 transcripts were reduced only by GEN-DEHP mixture in adult rat offspring, indicating a predominantly inhibitory effect of GEN on Sertoli cells (Jones et al., 2014; Jones et al., 2015). Additionally, MEHP treatment had an adverse effect on prepubertal Sertoli cell development in rat testes via stimulating Sertoli cell apoptosis and oxidative damages, and this effect could be partially counteracted by GEN through antioxidative action (Zhang et al., 2017). Several animal studies confirmed that Sertoli cells showed abnormal testicular phenotypes when mice or rats were exposed to GEN and DEHP mixtures (Xie et al., 2014; Walker et al., 2020). A recent study showed that MEHP at 0.1 μmol/L induced mitochondria swelling in Sertoli cells after 4 days of exposure, while no alterations in normal ultrastructure were observed following the GEN and DEHP combination (Zhang et al., 2022). Furthermore, Sertoli cells were found to be altered by MEHP in cultured human fetal testes, where MEHP treatment decreased the expression of Amh, a characteristic marker of Sertoli cells (Lambrot et al., 2009).

Compelling human data were reported in the analyses of maternal blood samples of pregnant women and infant in the Hokkaido Study. Looking at reproductive hormones, this epidemiological study unveiled a robust correlation between DEHP exposure *in utero* and impaired Sertoli and Leydig cell functions in male offspring (Araki et al., 2014). The study reported that *in utero* DEHP exposure reduced the concentration of Inhibin B, a hormone produced by Sertoli cells, in a dose-dependent manner, indicating an adverse effects of maternal phthalate exposure on Sertoli cell function in male infants (Araki et al., 2014).

Collectively, these findings strongly suggest that GEN and DEHP/MEHP mixtures have negative effects on Sertoli cell differentiation and function, with the predictable consequence of jeopardizing spermatogenesis. However, more investigations are needed to understand whether Genistein and DEHP, including their mixtures, affect macrophages - Sertoli cell interactions, that could contribute to male infertility.

## 5 Discussion

Testicular macrophages (tMΦ) are critical in maintaining immunological privilege in mammalian testis and contribute to tissue homeostasis, organogenesis, and normal testicular function via interactions with several types of testicular cells. Testicular development and function have an absolute requirement for androgens and estrogens, which are produced in a tightly regulated spaciotemporal way. Thus, these processes are prime targets of EDCs that disrupt androgen and estrogen homeostasis. The phytoestrogen GEN and the anti-androgenic phthalates DEHP and its metabolite MEHP were shown to hamper the development and function of critical testicular cell types in fetal to adult offspring, following perinatal exposures. We propose that these deleterious effects might be due to GEN, DEHP/MEHP or their mixtures interfering with the interactions between tMΦ and germ cells, Sertoli cells, or Leydig cells, at fetal to juvenile ages, thereby adversely affecting the development, homeostasis, and function of these cells and ultimately leading to male infertility.

Since infants can be exposed via maternal diet or baby formula to GEN in early life, and DEHP is ubiquitous, leaching out from consumer products, medical devices, cosmetics and other man-made products, early exposure to GEN and DEHP have high chance to happen and exert adverse effects on testicular function. GEN and DEHP may alter testicular developmental programs and functions by disrupting cell interactions in the testis. Moreover, mixtures may have unique effects not predicted by toxicological studies using single compounds (see Table 2). At present, there is clear evidence of tMΦ interactions with SSCs and Leydig cells, and the importance of these interactions for testicular function. The question remains about the possibility that tMΦ may interact directly or indirectly with Sertoli cells, as they do with SSCs, by communicating across the basement membrane of seminiferous tubules. We hypothesize that EDCs could directly target tMΦ in fetal to juvenile testis, disrupting their interactions with germ cells, Leydig cells and Sertoli cells, which would lead to impaired testicular functions. Intercellular communication depends on the secretion of various growth factors and signaling molecules. The interaction between tMΦ and SCCs has been shown to rely on the CSF1/CSF1R pathway. Thus, the EDC-driven dysregulation of CSF1 production in testicular macrophages could hamper SSC pool maintenance. Taken CSF1 as example, one could determine its expression levels using *in vitro* settings (e.g. organ on a chip; cell models) to screen novel EDCs with suspected male reproductive toxicity. One may also be able to use CSF1 or CSF1R downstream molecules for diagnostics purpose. Lastly, if CSF1 reduction is confirmed as one of the culprits in the adverse effects of EDCs, it could be used as target for the development of novel therapeutic agents to treat cases of deficient spermatogenesis or infertility, by researching pharmacological ways to palliate or prevent CSF1 decrease in testis and restore or support tMΦ-SSCs interactions.

**TABLE 2:** Summary of reported effects of GEN-DEHP/MEHP mixtures on testicular cells in mammalian species. tMΦ: testicular macrophages; GCs: germ cells; SCs: Sertoli cells; Leydig cells: LCs.

Cell type	Species	Main results	Study type	Ref
**TMΦ**	Rat	Increased mRNA expression of macrophage markers	*In vivo*	Walker et al. (2020)
**GCs**	Rat	Altered mRNA expression of undifferentiated GC (Thy1, Sox17) and differentiated (Kit, Sohlh2) markers	*In vivo*	Jones et al. (2014)
**GCs**	Rat	Altered mRNA expression of undifferentiated GC markers Plzf, Foxo1; differentiated GC marker Sohlh2	*In vivo*	Jones et al. (2015)
**SCs**	Rat	Altered mRNA expression of Wt1, Abp	*In vivo*	Jones et al. (2014)
**SCs**	Rat	Altered mRNA of differentiated SC gene marker Abp	*In vivo*	Jones et al. (2015)
**SCs**	Rat	Induced abnormal testicular phenotypes	*In vivo*	Walker et al. (2020)
**SCs**	Rat	GEN partially attenuated DEHP-induced SC toxicity	*In vivo*	Zhang et al. (2017)
**SCs**	Mouse	Induced histological abnormality (tubular vacuolation)	*In vivo*	Xie et al. (2014)
**LCs**	Rat	Altered expression of Hsd3b, Anxa1, Foxa3, and Pdgfra	*In vivo*	Jones et al. (2014)
**LCs**	Rat	Altered expression of Cyp11a1, Hsd3b	*In vivo*	Jones et al. (2015)
**LCs**	Rat	GEN normalized DEHP-induce testosterone changes	*In vivo*	Jones et al. (2015)
**LCs**	Rat	Long-term alterations in adult Leydig cell function	*In vivo*	Walker et al. (2020)
**LCs**	Mouse	Decreased gene expression of Insl3	*In vitro*	Jones et al. (2016)
**LCs**	Mouse	Altered steroid production and lipid homeostasis	*In vitro*	Jones et al. (2016)

## 6 Conclusion

**FIGURE 1:**
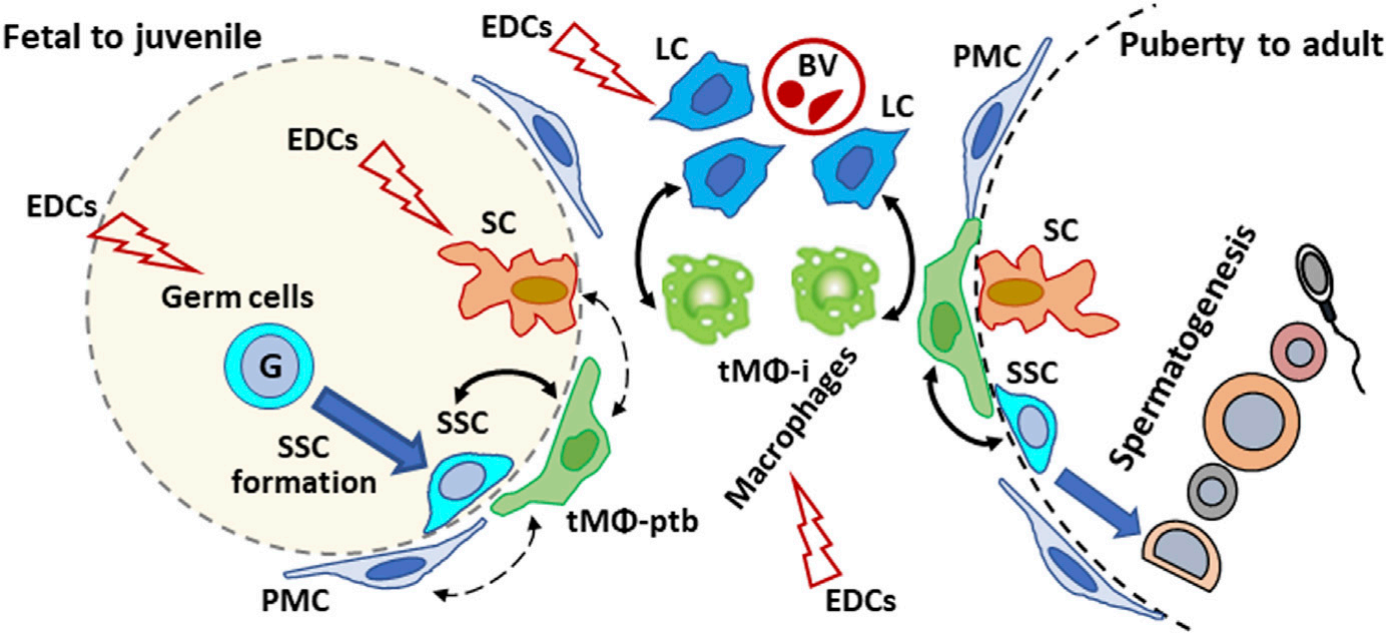
Schematic illustration of the proposed effects of endocrine disrupting chemicals (EDCs) on testicular cells interactions in mammalian testis from development to adulthood. Plain arrows: Direct interactions with macrophages. Dotted arrow: indirect/unreported interactions with macrophages. G: gonocyte; SSC: spermatogonial Stem Cell; tMΦ: testicular macrophages; tMΦ-ptb: peritubular tMΦ; tMΦ-i: interstitial tMΦ; PMC: peritubular myoid cells; SCs: Sertoli cells; Leydig cells: LCs; BV: blood vessels.

EDC can interfere with sex hormone homeostasis, disrupt the factors produced by testicular cells and their interactions with cells. Although tMΦ, SSCs, SCs, and Leydig cells have been the subject of experimental studies using *in vivo*, organ cultures or single-cell models to determine EDCs risk on male reproduction, information is lacking on whether disrupting cell-cell interactions could affect male reproductive functions and fertility. Future studies should examine the effects of EDCs such as GEN and DEHP, including mixtures at doses meaningful for human exposures and doses judged as non-toxic from single toxicant studies, on the interactions between macrophages and other testicular cell types. Moreover, studies mentioned in this review highlight the fact that determining reproductive risk based on a single EDC compound may not provide an adequate prediction of the reproductive toxicity of EDC mixtures to which humans are exposed. Comparing the impact of EDCs, individually and in mixtures, on testicular cell interactions should provide more insight into the toxicological mechanism of EDCs in male reproduction. As tMΦ are at the center of intricate cell-cell interactions (see Figure 1), encompassing multiple signaling molecules and signaling pathways, a first step could be to identify genes and signaling pathways dysregulated by EDCs in association with abnormal tMΦ functions, disrupted spermatogenesis and/or steroid production. Then, one could determine if treating tMΦ with EDCs would influence the behavior and functions of the other cell types, using *in vitro* approaches.

The ultimate goal of this strategy is to identify new targets of EDCs that could help in preventing testicular dysgenesis syndrome, by providing ways to assess risk more accurately, generate early interventions models to reduce the risk of hypospadias, infertility and testicular cancer. Ideally, this could lead to developing new diagnostic tools and/or therapies focusing on reestablishing healthy cell-cell interactions in the testis.
